# Oculopharyngeal Muscular Dystrophy: From Molecular Pathogenesis to Gene Therapy

**DOI:** 10.1007/s12035-026-06251-7

**Published:** 2026-10-13

**Authors:** Claudia Alberti, Delia Gagliardi, Dario Ronchi, Michela Ripolone, Laura Napoli, Maurizio Moggio, Giacomo Pietro Comi, Stefania Corti

**Affiliations:** 1 Centro Dino Ferrari, Department of Pathophysiology and Transplantation, University of Milan, Via Francesco Sforza 35, Milan, 20122 Italy; 2 Neurology Unit, Fondazione IRCCS Ca’ Granda Ospedale Maggiore Policlinico, Milan, Italy; 3 Neuromuscular and Rare Diseases Unit, Fondazione IRCCS Ca’ Granda Ospedale Maggiore Policlinico, Milan, Italy

**Keywords:** Oculopharyngeal muscular dystrophy, PABPN1, Polyalanine expansion, Intranuclear inclusions, Gene therapy, Dysphagia

## Abstract

Oculopharyngeal muscular dystrophy (OPMD) is a rare, late-onset, autosomal dominant muscular dystrophy caused by a trinucleotide GCN repeat expansion in the *PABPN1* gene on chromosome 14q11.2. The disease is clinically characterised by progressive ptosis, dysphagia, and proximal limb weakness, with onset typically between the fifth and sixth decades. OPMD affects approximately 1:100,000 individuals in Europe, with substantially higher prevalence in specific founder populations, reaching 1:600 among Bukharan Jews and 1:1,000 in French Canadians. The expanded polyalanine tract in PABPN1 promotes protein misfolding and formation of tubulofilamentous intranuclear inclusions, the histopathological hallmark of the disease. Pathogenic mechanisms involve a complex interplay of gain-of-function toxicity from aggregated mutant PABPN1, loss-of-function effects due to reduced polyadenylation efficiency, and dominant-negative impairment of poly(A) tail elongation, mRNA processing and nuclear mRNA export. Recent advances in proximity proteomics have refined the understanding of how the alanine expansion destabilises PABPN1 and alters its interactome. Diagnosis relies on molecular genetic testing of the *PABPN1* GCN repeat expansion, while PABPN1 immunofluorescence on muscle biopsy provides a highly sensitive and specific ancillary tool. Current management remains symptomatic, including cricopharyngeal myotomy for dysphagia and surgical correction of ptosis. The therapeutic landscape is evolving, with BB-301, an adeno-associated virus-based gene therapy employing a DNA-directed RNA interference (ddRNAi) silence-and-replace strategy, currently in a Phase 1b/2a clinical trial*.* This review provides a comprehensive synthesis of OPMD, spanning historical background, molecular genetics, pathophysiology, clinical presentation, diagnostic approach, and the emerging therapeutic pipeline.

## Introduction

Oculopharyngeal muscular dystrophy (OPMD, OMIM 164300) is a rare genetic disorder that affects the neuromuscular system. It occupies a unique position at the interface of the polyalanine expansion disorders, the muscular dystrophies, and the RNA-binding protein disorders. OPMD was first described in French-Canadians by Taylor in 1915 and was defined clinically as a specific syndrome by Victor, Hayes, and Adams in 1962. Over the last three decades, this condition has emerged as a model for explaining how rather small trinucleotide repeats may lead to the emergence of late-onset muscular degeneration [1–3].

The genetic basis of OPMD was first clarified in 1998 by Brais et al., where they were able to demonstrate that small expansions in the GCG repeat in the poly(A)-binding protein nuclear 1 gene (*PABP2*, current official name is PABPN1) on chromosome 14q11.2 were responsible for OPMD [4]. Unlike in polyglutamine disorders, which comprise a group of diseases characterised by CAG repeat expansions encoding elongated polyglutamine tracts (including Huntington disease and several spinocerebellar ataxias), expansions in the *PABPN1* gene in patients with OPMD are surprisingly small, adding only 1–8 alanine residues to the normal 10 alanine tract. Despite their small size, these expansions have significant effects on protein conformation and ultimately on skeletal muscle. OPMD is traditionally estimated to affect approximately 1 in 100,000 individuals in the general Caucasian population; however, its prevalence varies substantially across ethnic and geographic groups because of well-documented founder effects. The highest prevalence has been reported in the Bukharan Jewish population in Israel, at a rate of 1 in 600. The second highest prevalence is in French-Canadian kindreds in Quebec, Canada, at a rate of 1 in 1000. This high prevalence in the French-Canadian kindreds was traced to a founder mutation resulting from the limited number of settlers who came to Canada from France [5–8]. Other founder effects have also been reported in Hispanic kindreds in the southwestern United States, in the Canary Islands, in Mexico, in Uruguay, and in the Sakha Republic(Yakutia) [9–14].

To date, OPMD has been reported in more than 30 countries. However, the widely cited prevalence estimate of 1 per 100,000 individuals is unlikely to capture the marked geographic variability of the disease, particularly in populations with a founder effect. For example, in the Canary Islands, the prevalence has been reported to be at least 5.65 per 100,000 inhabitants [9]. Such findings suggest that OPMD remains substantially underdiagnosed in many settings.

This, combined with the late age of onset and insidious progression of the disease, probably explains the high proportion of cases of the condition that are undetected. Indeed, another reason is that symptoms of the disease often mimic those of other neuromuscular diseases, like myasthenia gravis and mitochondrial diseases.

Throughout the last decade, there has been a significant acceleration in the development of therapeutic strategies for the disorder. This assertion is supported by preliminary data indicating the effectiveness of gene therapy in managing the disorder. The first human trial for the evaluation of BB-301, a molecule created by Benitec Biopharma, has been started. The molecule BB-301 makes use of an adeno-associated virus delivery system to silence endogenous *PABPN1*, both the wild-type and the expanded allele, and to supply a codon-optimised, RNAi-resistant version of the wild-type *PABPN1* gene. These data were derived from the results of the Phase 1b/2a clinical trial evaluating BB-301 in patients with OPMD (NCT06185673) [15].

Understanding OPMD has implications that extend beyond the disease itself. As the only polyalanine expansion disorder affecting a non-transcription factor protein with late-onset muscle selectivity, OPMD has been used as a paradigm to study how subtle mutations in ubiquitously expressed RNA-processing factors lead to tissue-specific degeneration. Furthermore, the overlap between the molecular pathology of OPMD and physiological muscle ageing places this disease at a unique interface between genetic and age-related muscle wasting, which sheds light on the problem of sarcopenia as a whole. The development and early clinical evaluation of the silence-and-replace gene therapy approach to this disease process has implications for treating a vast range of autosomal dominant diseases caused by proteins that are both toxic and have dominant-negative properties.

The purpose of this narrative review is to present an updated overview of current knowledge about OPMD. The review covers the major landmarks in the history of OPMD from its discovery to molecular genetics, pathogenesis, phenotype, diagnostics, animal models, and treatment.

## Search Strategy and Selection Criteria

This narrative review was based on a literature search conducted in PubMed up to August 2026. Search terms included “oculopharyngeal muscular dystrophy”, “OPMD”, “PABPN1”, “polyalanine expansion”, “intranuclear inclusions”, “gene therapy”, “BB-301”, “dysphagia”, and related keywords. Priority was given to original research articles, clinical studies, translational investigations, landmark historical reports, and recent reviews relevant to the genetics, pathophysiology, diagnosis, and treatment of OPMD. Articles not directly related to OPMD or lacking sufficient methodological detail were excluded. Information regarding ongoing clinical trials was obtained from ClinicalTrials.gov and publicly available sponsor communications when peer-reviewed publications were not yet available. Data derived from non-peer-reviewed sources, including company press releases, are clearly identified in the text and should be considered preliminary until confirmed by peer-reviewed publication.

## Historical Perspective

The clinical recognition of OPMD has been a gradual process spanning more than one century. The first description of familial disease with ptosis and dysphagia is attributed to Taylor (1915), who reported the clinical findings in members of a French-Canadian family [2]. The condition remained poorly defined until 1962, when Victor, Hayes, and Adams published their landmark description in the New England Journal of Medicine of a family with nine affected members across three generations, establishing the cardinal clinical features and autosomal dominant inheritance pattern [3].

A critical advance in pathological characterisation came in 1980, when Tome and Fardeau described distinctive intranuclear inclusions (INIs) in skeletal muscle fibres using electron microscopy. These 8.5-nm tubulofilamentous structures became the pathological hallmark of the disease and provided the first clue to the intranuclear nature of the pathogenic process [16]. The molecular characterisation of the disease began with mapping the disease locus at chromosome 14q11.2-q13 [17]. The identification of short GCG repeat expansions in the *PABP2* gene, now known as *PABPN1*, as the causative mutation of the disease, published three years later, was a landmark study [4]. The demonstration of the involvement of the latter gene product, PABPN1, as a component of the INIs, reported by Calado et al. as early as 2000, established a direct correlation between the mutation and the histopathological hallmark of the disease [18].

## Molecular Genetics of OPMD

### The PABPN1 Gene and Its Protein Product

**Fig. 1:**
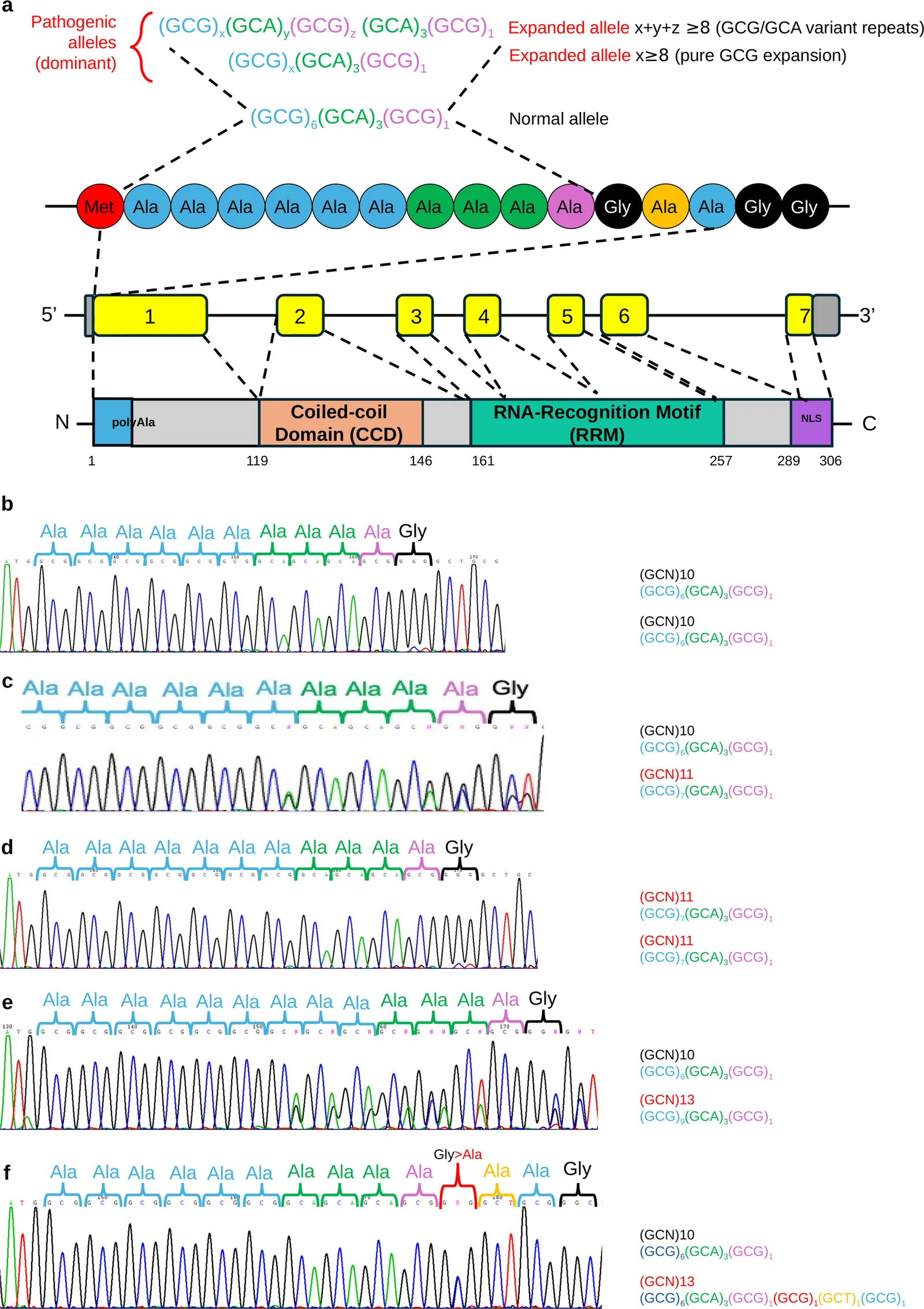
Genomic and protein architecture of PABPN1 and representative electropherograms of normal and pathogenic alleles. **a** Scheme of the PABPN1 gene (exons are numbered) and the encoded protein with the identification of specific protein domains. The amino acids in the surrounds of the poly-alanine stretch (polyAla) are highlighted. The structure of the GCG/GCA patterns corresponding to normal and expanded alleles is indicated; the repeat-length thresholds shown refer to fully penetrant alleles, whereas the (GCN)11 allele illustrated in **c** and **d** is pathogenic with reduced penetrance when heterozygous, as in **c**, and causes autosomal recessive disease when homozygous, as in **d**. **b**–**f** Electropherograms showing the sequence of the polyAla region (PABPN1, exon 1) in different subjects: **b** normal (GCN)10 homozygous allele; **c** pathogenic (incomplete penetrance, delayed onset) genotype displaying heterozygous (GCN)11 expanded allele; **d** pathogenic (GCN)11 homozygous allele; **e** pathogenic genotype displaying heterozygous (GCN)13 expanded allele; **f** pathogenic genotype displaying the heterozygous c.35G > C substitution resulting in the p.Gly12Ala aminoacidic change which elongates the polyAla stretch, (GCN)13, in the pathological range. In panels **c** and **e** the brackets above the trace follow the expanded allele (in panel **c** the trace starts at the second codon); at the codons where the two alleles differ (from the seventh codon onwards) the trace shows superimposed peaks

The *PABPN1* gene (reference transcript NM_004643.4) is located on chromosome 14q11.2 and codes for a nuclear protein containing 306 amino acids, which plays a crucial role in the mRNA polyadenylation process and post-transcriptional gene expression. The protein contains four domains: the polyalanine domain (aa 2–11), the coiled-coil domain that increases the activity of poly(A) polymerase (aa 119–146), the RNA recognition motif domain that enables protein–protein and RNA binding (aa 161–257), and the nuclear localisation signal at the carboxyl terminal of the protein (aa 289–306) *(*Fig. 1*)* [19]. Although the calculated molecular weight of PABPN1 is 32.8 kDa, the observed molecular weight is 50 kDa during protein electrophoresis under reducing conditions, an anomalous mobility. PABPN1 is also subject to post-translational modification, in particular arginine methylation of its C-terminal region by type I protein arginine methyltransferases, including PRMT1 and PRMT3, which are themselves sequestered, together with HSP70, within the intranuclear inclusions formed by expanded PABPN1 [20]. The protein is expressed ubiquitously, yet OPMD affects specific groups of skeletal muscle cells. This tissue specificity of OPMD’s effects on muscle cells remains partially explained. The protein PABPN1 is a shuttle protein that localises to the nucleus and cytoplasm. The protein plays a role in various aspects of RNA metabolism, such as control of poly(A) tail length, regulation of alternative polyadenylation, processing of long non-coding RNAs and small nuclear RNAs, and nuclear mRNA surveillance/export [19, 21].

### OPMD Genetics

The disease-causing mutation involves an expansion of the GCN trinucleotide repeat sequence found in the first exon of the *PABPN1* gene. The use of “GCN” to refer to the trinucleotide repeat is justified by the codon degeneracy of alanine: GCG and GCA predominate within the repeat tract, although all four alanine codons have been reported [13]. The normal allele harbours 10 GCN repeats with the canonical structure (GCG)6(GCA)3(GCG)1 encoding a polyalanine tract of 10 residues [4]. Fully penetrant pathogenic alleles contain 12 to 18 alanine residues and can arise through different mutational mechanisms. The most common are pure GCG expansions, but pathogenic alleles with GCA variant repeat have also been described, in which the total alanine count is 12 or more through various combinations of GCG and GCA codons [22]. For clinical purposes, the total number of contiguous alanine residues, rather than the specific nucleotide sequence, determines pathogenicity.

A rare point mutation (p.Gly12Ala) that extends the polyalanine tract without altering the repeat number has also been identified [23, 24]. Recently, a single nucleotide variant resulting in the p.Gly12Trp change has been identified in a suspected OPMD patient [25] and currently represents the only exception to the pathogenic expansion/elongation of the alanine stretch as the molecular driver of disease.

The mutational spectrum of *PABPN1* expansions is heterogeneous. Analysis of 86 patients carrying a *PABPN1* expansion, including three compound heterozygotes, identified 13 different expansion types, six of which combine GCA and GCG codons and almost all of which are consistent with a mutational mechanism of unequal recombination [26]. This mechanism had originally been proposed on the basis of duplicated alleles in Japanese patients that could not be explained by simple expansion of GCG repeats or by DNA slippage [27]. The meiotic and mitotic stability of OPMD expansions contrasts sharply with the instability observed in polyglutamine disorders, where dynamic repeat instability drives anticipation across generations. In OPMD, disease severity is primarily determined by the static repeat length rather than by intergenerational expansion, and anticipation is not a recognised feature.

Approximately 90% of OPMD cases carry one normal (GCN)10 allele and one expanded allele containing (GCN)11 to (GCN)18 repeats, while the remaining 10% harbour biallelic expansions, either compound heterozygosity, in which a (GCN)11 allele is combined with a second expanded allele, or homozygosity for smaller expansions, in particular (GCN)11, (GCN)12 and (GCN)13 alleles [13, 28]. Patients harbouring biallelic expansions (GCN) ≥ 12 seem to develop an earlier onset of symptoms (< 35 years of age), more aggressive clinical progression (with extramuscular features including mild cognitive impairment and psychiatric symptoms) and reduced lifespan (within the sixth decade), mimicking clinical presentations associated with heterozygous (GCN)18 alleles, the maximum number of GCN so far reported in the locus. This again points to a dosage effect of the expanded alleles, showing incomplete dominance.

The distinction between dominant and recessive alleles of the gene plays an important role. Heterozygous individuals carrying an expansion of (GCN)12 or greater develop OPMD with age-dependent penetrance, which is essentially complete after 70 years of age. Biallelic (GCN)11 genotypes, by contrast, underlie the autosomal recessive presentation of the disease.

#### The (GCN)11 Allele

The (GCN)11 allele, encoding 11 alanines, has a prevalence of 1–2% in populations of European, North American, and Japanese descent [1]. Initially classified as a benign polymorphism, this allele exhibits three distinct modes of pathogenicity. In the homozygous state, (GCN)11/(GCN)11 causes autosomal recessive OPMD [29]. In compound heterozygosity with a larger expansion, it acts as a disease-modifying allele that accelerates clinical progression. Finally, a minority of single (GCN)11 carriers develop a mild, late-onset form, indicating that the allele can also behave as dominant with markedly reduced penetrance [30].

#### Genotype–Phenotype Correlations

The largest study on genotype–phenotype correlations was carried out by Richard et al. on 354 French index patients. They reported a statistically significant inverse correlation between the sizes of expansions and age at diagnosis [28]. Clinical variability is further influenced by zygosity: homozygotes and compound heterozygotes for (GCN) ≥ 12 alleles exhibit earlier onset, faster dysphagia progression, and greater functional impairment [31, 32]. Despite these correlations, substantial intra- and interfamilial variability persists, suggesting additional genetic and environmental modifiers remain to be identified.

OPMD affects males and females equally and no difference in parental transmission of the two alleles has been demonstrated.

While (GCN)11 alleles are associated with incomplete penetrance, age-dependent full penetrance is estimated for (GCN)12–18 alleles. For example, fewer than 10% of (GCN)13 carriers are symptomatic by 49 years of age, rising to 31% at 50–59 years and 63% at 60–69 years, with essentially complete penetrance after 70 years of age [13].

As a consequence, the identification of OPMD patients likely entails the existence of additional patients in the proband’s pedigree requiring appropriate genetic and clinical counselling.

### OPMD in the Context of Polyalanine Expansion Diseases

OPMD belongs to a group of nine human diseases caused by polyalanine tract expansions [33]. In the human proteome, 494 annotated proteins harbour 604 polyalanine domains longer than four alanine residues, and 124 of these proteins carry a domain exceeding seven alanines [34]. No polyalanine domain longer than 20 residues has been identified in the human, *Drosophila* or *C. elegans* genomes. A distinguishing feature of OPMD within this disease group is that PABPN1 is the only causative protein that is not a transcription factor involved in neurodevelopment. All other polyalanine expansion diseases manifest prenatally or in early childhood, whereas OPMD is a late-onset disease affecting a ubiquitously expressed RNA-processing factor, making it a unique paradigm for understanding polyalanine-mediated pathology in post-mitotic tissues. Additional peculiar aspects include the following: atypical location of polyalanine tract (exon 1, after the start codon), very tight mutational threshold (10 GCN: normal; 11 or more GCN: pathological with variable penetrance) and accumulation of intranuclear inclusions in patients carrying expansions. These elements further differentiate OPMD from other polyalanine disorders and overlap pathological aspects observed in neurological disorders due to polyglutamine expansions.

## Pathophysiology

### Protein Misfolding and Intranuclear Inclusions

The extended polyalanine tract induces protein misfolding, which results in the formation of oligomers that aggregate further to form the 8.5-nm tubulofilamentous structures of INIs. Calado et al. showed that INIs contain aggregated PABPN1, poly(A) RNA, ubiquitin, and proteasomal subunits [18]. Subsequent reports identified other components, including the heat shock protein HSP70, protein arginine methyltransferases (PRMT1, PRMT3), which interact with and methylate PABPN1 and have been localised in INIs, the autophagy-related protein p62, and the endoplasmic reticulum (ER) chaperone GRP78/BiP, which has been localised in INIs, but it is likely a reflection of the activation of the ER stress response pathway rather than a specific stoichiometry in INIs [20, 35]. INIs are typically found in 2–15% of myonuclei [35], with frequency increasing with age, paralleling the clinical course.

Roth and colleagues conducted the most comprehensive characterisation of PABPN1 aggregates to date on a large multinational collection of 90 frozen muscle biopsies from 73 OPMD patients with known genotype and age. This study demonstrated that both age and genotype influence PABPN1 aggregates: the percentage of myonuclei containing aggregates increases with age, whereas the frequency of HSP70 co-localisation with aggregates increases with expansion size. Interestingly, pharyngeal muscles, which are the most clinically affected, contain fewer aggregates than the other muscles sampled in the same patients, possibly reflecting the lower PABPN1 content and greater regenerative activity of this muscle. The study also generated a xenograft model by transplanting human OPMD muscle into immunodeficient mice, demonstrating that PABPN1 aggregates reappear rapidly after muscle regeneration, albeit at a lower level than before grafting, with important implications for cell-based therapies [35].

The relationship between INIs and cellular toxicity remains debated. Although it is clear that aggregates correlate with disease, there is some suggestion that they may be a partly protective cellular response, sequestering misfolded protein, with the soluble oligomeric species being the main toxic species. Fluorescence recovery after photobleaching (FRAP) experiments have shown that PABPN1 molecules are not irreversibly trapped within inclusions but rather exchange dynamically between the aggregated and soluble pools, indicating that the inclusions are in equilibrium with the surrounding nucleoplasm rather than constituting static, inert deposits [36]. Time-lapse imaging has further identified nuclear speckles as the sites where PABPN1 aggregates nucleate and grow, progressively depleting these compartments of PABPN1 and poly(A) RNA [37].

### Gain-of-Function Mechanisms

Several lines of evidence support a toxic gain-of-function model. Transgenic mouse models expressing expanded PABPN1 develop muscle pathology and aggregates even in the presence of normal endogenous *Pabpn1* [38, 39]. Aggregates sequester essential nuclear factors, including components of the splicing and polyadenylation machinery. Evidence from *Drosophila* models demonstrates that toxicity requires the RNA-binding function of PABPN1, indicating that interaction between aggregated PABPN1 and RNA targets contributes to cellular damage [40].

### Loss-of-Function Mechanisms

Sequestration of both mutant and wild-type PABPN1 within aggregates reduces the functional pool available for essential RNA-processing activities. This compromises the efficiency of polyadenylation, leading to a shift in proximal poly(A) site usage, reducing 3ʼ untranslated regions, and disrupting the expression of critical muscle-related genes [41, 42]. Heterozygous loss of *Pabpn1* in mice causes mild muscle pathology, shortened poly(A) tails, and mitochondrial dysfunction, supporting the contribution of loss-of-function to disease [43].

### Dominant-Negative Effects and Impaired RNA Export

Recent work using TurboID proximity labelling in differentiated C2C12 myotubes has provided new insights. Mezzell et al. found that in cells expressing Ala17 PABPN1, there was an increased interaction with RNA splicing/polyadenylation and ubiquitin–proteasome components. Moreover, they found that PABPN1 with an Ala17 expansion had reduced interaction with TREX complex components, which are required for RNA export. This caused nuclear retention of polyadenylated RNA in cells expressing PABPN1 with an Ala17 expansion, even in the presence of wild-type PABPN1 [44]. Furthermore, PABPN1 with an Ala17 expansion was more insoluble and had a faster degradation rate of its soluble fraction compared with wild-type (Ala10) PABPN1. Oligo d(T) fluorescence in situ hybridisation (FISH) confirmed nuclear poly(A) RNA retention in Ala17-expressing myotubes, and in primary myotubes from the knock-in +/Ala17 mouse model, providing a mechanistic link between the dominant-negative effect and downstream RNA export dysfunction. This study provides evidence that PABPN1 with an alanine expansion can function abnormally in cells with or without wild-type PABPN1. This suggests that both loss-of-function and dominant-negative effects contribute to OPMD pathology. In conclusion, current evidence supports a model in which gain-of-function toxicity, loss-of-normal PABPN1 function, and dominant-negative impairment of RNA export are all involved in causing progressive muscle degeneration in OPMD. This model is important because it suggests that an effective treatment strategy to combat OPMD pathology needs to target both gain-of-function toxicity and loss-of-normal PABPN1 function [19].

### Mitochondrial Impairment

**Fig. 2:**
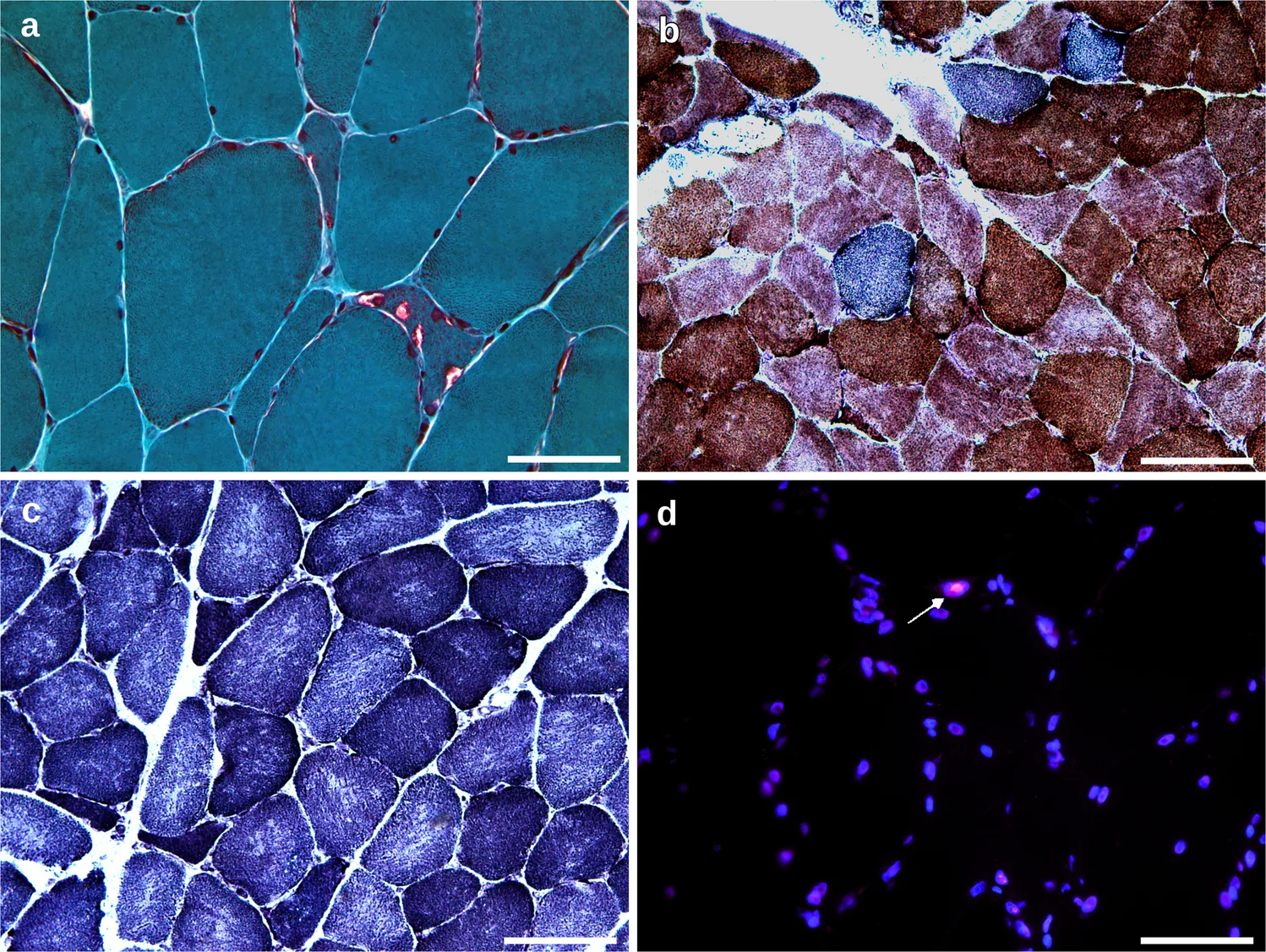
Histopathological and immunofluorescence features of skeletal muscle in oculopharyngeal muscular dystrophy. Cryostat sections of skeletal muscle from genetically confirmed OPMD cases were processed for routine histological and enzyme histochemical staining and for PABPN1 immunofluorescence. **a** Modified Gomori trichrome staining shows rimmed vacuolar change in an atrophic fibre, a finding that is typical, although not specific, of OPMD. **b** Combined cytochrome c oxidase/succinate dehydrogenase (COX/SDH) double staining reveals three COX-negative, SDH-positive fibres (blue), pointing to a mild secondary mitochondrial dysfunction that has been repeatedly reported in OPMD muscle. **c** NADH-tetrazolium reductase staining highlights an irregular, moth-eaten intermyofibrillar pattern with pale areas in some fibres. **d** Immunofluorescence performed after KCl pre-treatment, using a monoclonal anti-PABPN1 antibody (Abcam ab75855, clone EP3000Y) detected with a Cy3-conjugated goat anti-rabbit secondary antibody and counterstained with DAPI (blue), shows one nucleus with bright intranuclear accumulation of PABPN1 (arrow), corresponding to the insoluble inclusions that represent the histopathological hallmark of the disease, against a background of nuclei showing only weak, diffuse PABPN1 signal. Scale bars: 50 µm in **a**; 100 µm in **b** and **c**; 50 µm in **d**

Mitochondrial dysfunction has emerged as a recurrent feature of OPMD, although current evidence suggests that it is more likely a downstream consequence of PABPN1 dysfunction than the primary pathogenic event. Early studies reported mitochondrial abnormalities in muscle biopsies from OPMD patients, including cytochrome c oxidase-negative fibres (Fig. 2) and ultrastructural alterations such as paracrystalline inclusions and abnormal cristae [45–49]. These findings initially raised the possibility of a primary mitochondrial involvement but were later interpreted as markers of muscle degeneration rather than evidence of a mitochondrial disorder. More recent studies have provided a mechanistic explanation for these observations. Chartier et al., using *Drosophila* and mouse models, showed that mutant PABPN1 alters post-transcriptional RNA regulation by promoting Smaug (Smg)-dependent deadenylation. Smaug is an RNA-binding protein that recruits the CCR4–NOT deadenylase complex to shorten poly(A) tails and trigger degradation of specific mRNAs, particularly those encoding mitochondrial proteins [50]. As a result, mitochondrial biogenesis and oxidative metabolism are impaired, contributing to muscle weakness. Interestingly, reducing deadenylation improved muscle function despite increasing PABPN1 aggregates, suggesting that nuclear aggregates may not represent the primary toxic species, especially during the early stages of the disease. These findings are supported by knock-in mouse models, which exhibit early mitochondrial defects together with altered poly(A) metabolism, and by more recent multi-omics studies showing that PABPN1 aggregation disrupts RNA processing, mRNA export, and translation, ultimately affecting cellular metabolism [43, 51]. Overall, current evidence supports a model in which mitochondrial dysfunction arises secondary to defects in RNA metabolism caused by mutant PABPN1 and represents a major contributor to disease progression rather than the initiating pathogenic event.

### OPMD and Ageing: Overlapping Mechanisms

An intriguing aspect of OPMD pathophysiology is its overlap with the biology of muscle ageing. PABPN1 levels naturally decline in skeletal muscle with advancing age, and age-related changes in alternative polyadenylation patterns closely resemble those observed in OPMD [42]. The INIs themselves are also overrepresented in older individuals, and the pathways affected in OPMD, such as the ubiquitin–proteasome pathway, mitochondrial function, and insulin signalling, are all key components of the ageing process. This coincidence of effects has prompted the suggestion that OPMD is an accelerated form of ageing in skeletal muscle due to the PABPN1 mutation, with the expanded protein acting to worsen subclinical effects of ageing on protein homeostasis and RNA metabolism.

This theory of ageing is supported by transcriptomic studies that have shown that the gene expression changes seen in OPMD muscle are very similar to those seen in ageing muscle, including the down-regulation of mitochondrial genes and the up-regulation of extracellular matrix genes. Evidence suggests that wild-type PABPN1 may form non-pathogenic nuclear foci in aged post-mitotic muscle cells, suggesting that some degree of aggregation is part of normal ageing biology that is pathologically amplified by the polyalanine expansion. The fact that OPMD tends to manifest in the fifth or sixth decade of life, during which time the normal decrease in PABPN1 levels with age acts to accentuate the effect of the mutation, provides a rationale for the delayed onset. This model predicts that interventions targeting general ageing mechanisms, such as autophagy enhancement or mitochondrial protection, could have therapeutic value in OPMD even without directly addressing the PABPN1 mutation. Finally, the OPMD disease process could provide an accessible model system to investigate the process by which benign, age-related protein aggregation leads to disease-causing aggregation, an issue of general importance in neurodegenerative diseases such as Alzheimer’s and Parkinson’s diseases.

## Clinical Features

### The Cardinal Triad

The clinical features of patients suffering from OPMD are characterised by progressive ptosis, dysphagia, and proximal limb weakness. Ptosis is the primary symptom, appearing as bilateral and progressive lid drooping in the fifth or sixth decade of life, often accompanied by compensatory backward head tilt. External ophthalmoplegia, particularly limitation of upward gaze, is common. Jordan and colleagues demonstrated that Müller’s muscle shows dystrophic changes in 72.5% of biopsied OPMD patients, with fatty infiltration of the smooth muscle intermixed with intact muscle bundles [52].

Although ptosis and dysphagia represent the cardinal manifestations of OPMD, the clinical presentation at symptom onset is heterogeneous. While ptosis is frequently the first recognised symptom, a substantial proportion of patients may initially present with dysphagia [28]. Respiratory disease is the main cause of death, and aspiration pneumonia is a major complication [13, 53]. Weakness and atrophy of the tongue also cause dysphagia and dysarthria.

Proximal limb weakness develops later, typically in the sixth decade [53]. Lower limbs are more severely affected, with hip flexors being particularly vulnerable. Approximately 14% of patients require a wheelchair for long distances or daily living before the age of 75 [53]. Progression is slow over decades, and life expectancy is near normal, with a median age at death of 77 years in patients with (GCN)13 expansion, corresponding to approximately 23 years of survival after the median onset age of 54 years [53]. The slow progression and relative preservation of life expectancy distinguish OPMD from more rapidly progressive muscular dystrophies.

### Additional Features and Natural History

Beyond the cardinal triad, OPMD may present with dysphonia, nasal voice quality, facial weakness, and cognitive impairment affecting executive function, particularly with larger expansions [54]. A rare severe form has been described with onset before age 45, associated peripheral neuropathy, and more rapid progression. Kroon and colleagues documented a measurable decline in deltoid strength, tongue strength, and functional capacity over 20 months of follow-up in 43 patients, establishing sensitive outcome measures for clinical trials [55]. The study demonstrated that deltoid muscle strength, measured by fixed dynamometry, and the 10-step stair test are among the most responsive measures, making them valuable endpoints for intervention studies [55].

The natural history of OPMD follows a predictable pattern. This pattern can be divided into clinical phases. The first phase is the presymptomatic phase. This phase is characterised by the accumulation of intranuclear inclusions without any symptoms. This phase can last decades. The second phase is the early symptomatic phase. This phase is characterised by ptosis. This ptosis is usually thought to be caused by ageing. The third phase is the progressive phase. This phase is characterised by the involvement of the pharyngeal muscles, the proximal limb muscles, and finally the other muscles.

## Diagnostic Approach

### Genetic Testing

Genetic testing of the *PABPN1* GCN repeat expansion is the gold standard. Single-gene testing using targeted sizing by fragment analysis, repeat-primed PCR, or Sanger sequencing of *PABPN1* exon 1 provides a definitive diagnosis. Repeat primed PCR and Sanger sequencing might help to characterise the sequence of target regions looking for the presence of heterogeneous GCG/GCA combinations. However, as noted above, clinical presentation reflects the number of GCN repeats, irrespective of the presence of pure GCG repeats and GCA variant repeat. Complex sequence motifs might hamper the correct determination of allele size. Conversely, the detection of rare p.Gly12Ala/Trp alleles escapes fragment analysis and requires exon 1 sequencing. For atypical phenotypes, next-generation sequencing (NGS)-based approaches (including short-read methods) can identify the expansion, although the GC-rich repeat region may have low coverage and should be carefully checked in NGS scans [13].

### Muscle Biopsy and PABPN1 Immunofluorescence

Muscle biopsy is rarely required when genetic testing confirms the diagnosis, but it remains valuable in atypical cases. Characteristic findings include fibre size variation, rimmed vacuoles and small angulated fibres (Fig. 2), and, on electron microscopy, 8.5-nm tubulofilamentous INIs.

PABPN1 immunofluorescence following KCl pretreatment represents a significant diagnostic advance. Galimberti and colleagues demonstrated a sensitivity of 100% in genetically confirmed patients and a specificity of 96% in patients with other genetically proven neuromuscular diseases. Retrospective analysis of an undiagnosed neuromuscular disease cohort identified 23 new OPMD cases (10.4%), highlighting the extent of underdiagnosis [56]. The number of aggregates also correlated with the age of patients with identical genetic background [35], suggesting that aggregate load reflects disease duration more than expansion size. This assay therefore represents a histological marker reflecting OPMD progression in a prognostic setting and a potential quantitative outcome measure for interventional clinical trials.

In addition, p62 immunohistochemistry may provide a practical pathological marker in diagnostic settings where PABPN1 immunostaining is not routinely available. Ogasawara et al. demonstrated that p62-positive intranuclear inclusions are frequent in OPMD myonuclei and correspond to PABPN1-positive aggregates, supporting the utility of p62 staining in differentiating OPMD from OPDM and other rimmed vacuolar myopathies [57].

Several histopathological studies have reported mitochondrial abnormalities in skeletal muscle biopsies from patients with OPMD. These findings include cytochrome c oxidase (COX)-negative fibres, bizarre enlarged mitochondria with abnormal cristae and nonspecific myopathic changes in the absence of ragged-red fibres [45], abnormal mitochondria associated with fingerprint inclusions on electron microscopy [46], and paracrystalline or electron-dense mitochondrial inclusions within affected muscle fibres [47–49]. In muscle biopsies, mitochondrial abnormalities are generally regarded as secondary and nonspecific morphological findings and should not be interpreted as evidence of a primary mitochondrial disorder.

### Supportive Investigations

Serum creatine kinase is typically mildly elevated. Electromyography reveals myopathic changes in approximately 92% of patients [58]. Muscle MRI demonstrates progressive fatty infiltration of the tongue, adductor magnus, and soleus; quantitative MRI detects subclinical progression. Muscle ultrasound and videofluoroscopy complement the diagnostic workup and are increasingly used as endpoints in clinical trials [59].

### Differential Diagnosis

Late-onset ptosis combined with dysphagia and a positive family history with autosomal dominant inheritance is highly suggestive of OPMD. However, the differential diagnosis of the cause of the symptoms in the case of OPMD may be quite extensive, which could lead to the delayed diagnosis of the patient’s condition. The most common cause of misdiagnosis in the case of patients with OPMD is myasthenia gravis. Myasthenia gravis is a condition where the patient may experience symptoms that are transient, fatigable, and may also be positive for autoantibodies, which include the anti-AChR or anti-MuSK antibodies [13]. Some patients may be treated with immunosuppressive drugs before the actual cause of the symptoms is identified.

Patients with mitochondrial myopathies, such as chronic progressive external ophthalmoplegia, may also be misdiagnosed with the condition. The mitochondrial myopathies have ragged red fibres on the modified Gomori trichrome stain and multisystem involvement with sensorineural deafness, cardiac conduction defects, and diabetes. Myotonic dystrophy type 1 patients with the CTG repeat expansion in the *DMPK* gene may also be misdiagnosed with the condition. Myotonic dystrophy patients may have myotonia, predominantly distal muscle weakness, frontal balding, and cataracts [13]. Oculopharyngodistal myopathy (OPDM) has recently been recognised as a distinct entity caused by non-coding CGG repeat expansions in *LRP12*, *GIPC1*, *NOTCH2NLC*, and *RILPL1* (OPDM1–4), described mainly in Asian populations, and by a CCG expansion in the 5’ untranslated region of *ABCD3* (OPDM5) in individuals of European ancestry [60], and shows similar involvement of the pharynx and the eye but differs in the involvement of the distal limbs. Eura et al. compared muscle imaging in 54 patients with OPDM and 57 with OPMD, finding early soleus and medial gastrocnemius involvement in OPDM, whereas severe adductor magnus and gluteus medius/minimus involvement indicated OPMD; a tibialis anterior score equal to or greater than the adductor magnus score identified OPDM with 86.4% sensitivity and 100% specificity [61]. Sporadic inclusion body myositis is characterised by involvement of finger flexors or quadriceps in males over 50 years of age with inflammatory infiltrates. Congenital fibrosis of extraocular muscles is a non-progressive condition that is present at birth [13]. Of significance, just like OPDM, there exists genetic heterogeneity in the OPMD syndrome itself, exemplified by the discovery of OPMD2 (OMIM 620460). OPMD2 manifests due to heterozygous frameshift mutations in the last coding exon of the *HNRNPA2B1* gene, which abolish the native stop codon and extend the reading frame, generating a protein with a common C-terminal extension that mislocalises to the cytoplasm. OPMD2 differs from classic OPMD in the onset of symptoms during childhood or adolescence. In patients showing features of OPMD with early onset who do not have PABPN1 mutations, genetic screening for OPMD2 should be carried out [62].

Genetics pearl for practising neurologists: the presence of late onset ptosis and dysphagia in a patient with a family history of the condition warrants genetic testing for OPMD before muscle biopsy.

### Imaging Biomarkers and Emerging Omics Approaches

The development of sensitive and specific biomarkers is essential for monitoring disease progression and evaluating therapeutic responses in OPMD clinical trials. Muscle MRI techniques have become increasingly important, with fat fraction quantification techniques that detect fatty infiltration of muscle before any other signs of weakness. Tongue, adductor magnus, and soleus show the earliest and most severe involvement, establishing a characteristic pattern that aids diagnosis and staging. Dixon-based quantitative MRI and T2-weighted imaging provide reproducible metrics of fat infiltration and oedema that can serve as surrogate endpoints. Videofluoroscopic swallowing study (VFSS), scored with standardised scales such as the Dynamic Imaging Grade of Swallowing Toxicity (DIGEST) framework, the normalised residue ratio scale (NRRSv) and total pharyngeal residue (TPR), together with patient-reported instruments such as the Sydney Swallow Questionnaire (SSQ), are among the swallowing outcome measures available for OPMD [59]. Videofluoroscopic assessment of pharyngeal residue and the Sydney Swallow Questionnaire are among the outcome measures of the ongoing BB-301 study [15].

Transcriptomic studies have revealed dysregulation of the ubiquitin–proteasome system, insulin signalling, and genes related to cell structure, with substantial overlap between OPMD-specific and age-related transcriptomic changes. MicroRNA profiling has identified miR-200c and miR-451a as showing age-dependent increases in symptomatic patients. Interactome proteomics in skeletal muscle has shown that alanine-expanded PABPN1 associates with a larger set of proteins than the wild-type protein, and that the ALS/frontotemporal dementia (FTD)-associated protein TDP-43 co-immunoprecipitates preferentially with the expanded form, accumulating in high-molecular-weight and insoluble complexes with evidence of impaired TDP-43-dependent splicing [63], providing a molecular crosslink with ALS/FTD pathobiology that may offer insights into shared mechanisms of RNA-binding protein aggregation across neurodegenerative diseases. Cytokine profiling, metabolomics, and epigenetic studies further enrich the biomarker landscape. Integration of multi-omics datasets in longitudinal studies will be essential for identifying optimal monitoring biomarkers and designing efficient clinical trials [59].

## Animal Models

These have been critical in understanding the mechanisms of the disease and developing therapeutic interventions. Three principal mouse models have been generated. The first, developed by Hino and colleagues, employed the CAG (beta-actin) promoter to express 13-alanine expanded PABPN1 ubiquitously. This model reproduced muscle pathology and intranuclear aggregates but was confounded by premature death, growth retardation, and extra-muscular pathology not seen in human disease [38]. The second, generated by Dion and colleagues, used a putative *PABPN1* promoter to drive expression of expanded PABPN1 at more physiological levels. Unexpectedly, this model produced a primarily neuronal phenotype with coordination defects and peripheral neuropathy, but no skeletal muscle aggregates, likely due to insufficient expression levels in skeletal muscle [64].

The third and most widely used model, the Ala17 model characterised by Trollet and colleagues, employs the human skeletal alpha-actin promoter to drive expression of bovine 17-alanine expanded PABPN1 specifically in skeletal muscle. This model most closely recapitulates human OPMD, with progressive muscle weakness, intranuclear aggregates, muscle fibrosis, and molecular changes including dysregulated gene expression restricted to fast glycolytic fibres [39]. The Ala17 model has served as the primary preclinical platform for evaluating therapeutic strategies, including the silence-and-replace gene therapy that led to BB-301. However, it is important to note that this model relies on transgenic overexpression rather than knock-in of the expansion. Notably, Zhang and colleagues recently demonstrated for the first time impaired pharyngeal muscle function and satellite cell proliferation in the *Pabpn1* +/Ala17 knock-in model, providing evidence that defective basal autophagy in pharyngeal myoblasts contributes to the craniofacial selectivity of OPMD [65].

Knock-in models expressing Ala17-expanded *Pabpn1* from the native locus, generated by Vest and colleagues, revealed early-onset mitochondrial defects including altered cristae morphology and reduced oxidative phosphorylation complex levels [43]. These findings indicate that mitochondrial dysfunction may arise early during disease progression in this knock-in model. However, its early occurrence does not necessarily imply a primary pathogenic role, as mitochondrial impairment may develop downstream of the direct effects of mutant PABPN1 on RNA metabolism and cellular homeostasis. Comparison with heterozygous *Pabpn1* knockout mice showed partial phenotypic overlap, supporting the contribution of loss-of-function to disease, but also identified knock-in-specific phenotypes that cannot be explained by loss-of-function alone, confirming the multi-mechanism pathogenic model.

*Drosophila* models developed by Chartier and colleagues provided complementary insights, demonstrating that the RNA-binding domain of PABPN1, rather than the polyalanine tract alone, is required for muscle toxicity [40]. This finding has important implications for understanding disease mechanism, suggesting that expanded PABPN1 acquires enhanced ability to interact aberrantly with RNA targets, rather than simply forming inert aggregates. The collective evidence from multiple model systems indicates that alanine-expanded PABPN1 is intrinsically toxic and can produce muscle pathology in transgenic models that retain intact endogenous alleles [38–40], without excluding the concurrent loss-of-function and dominant-negative contributions discussed above.

## Current Management

Management remains symptomatic and multidisciplinary. Dysphagia management includes dietary modification, speech-language pathology interventions, cricopharyngeal myotomy (surgical or endoscopic), botulinum toxin injection, and percutaneous endoscopic gastrostomy in severe cases. Cricopharyngeal myotomy is the best documented of these interventions: in a retrospective series of 39 patients treated by extramucosal myotomy, symptoms remitted completely in 25 patients and improved markedly in 10, but dysphagia recurred in 12 of the 35 responders after a mean of 39 months [66]. Ptosis management is guided by levator function: aponeurotic advancement for good function (> 12 mm), combined levator-Müllerʼs advancement for intermediate function (8–12 mm), and frontalis sling suspension for poor function (< 8 mm). Undercorrection is preferred to avoid corneal exposure. Primary frontalis suspension may also be considered as the initial procedure in early-onset disease with moderate-to-severe ptosis, since it will generally be required at a later stage, although no universally accepted algorithm exists [52]. Proximal limb weakness is addressed through physiotherapy, occupational therapy, and assistive devices. Nutritional counselling, genetic counselling, and respiratory monitoring complete the multidisciplinary approach.

The psychosocial impact of progressive dysphagia in patients with OPMD needs to be emphasised. Mealtimes are a source of anxiety and social isolation. Choking episodes are a source of considerable distress to both patients and caregivers. Videofluoroscopic evaluation helps in assessing patients with progressive dysphagia and in adjusting dietary recommendations to prevent aspiration pneumonia. Patient registries such as the National Israeli Registry for OPMD (IsrO-PMD), a national prospective registry that collects standardised neurological, swallowing and ophthalmological data at periodic follow-up, are intended to document the natural history of the disease and to provide a basis for recruitment into future clinical trials [67].

## Emerging Therapeutic Strategies

### Gene Therapy: The Silence-and-Replace Approach

**Fig. 3:**
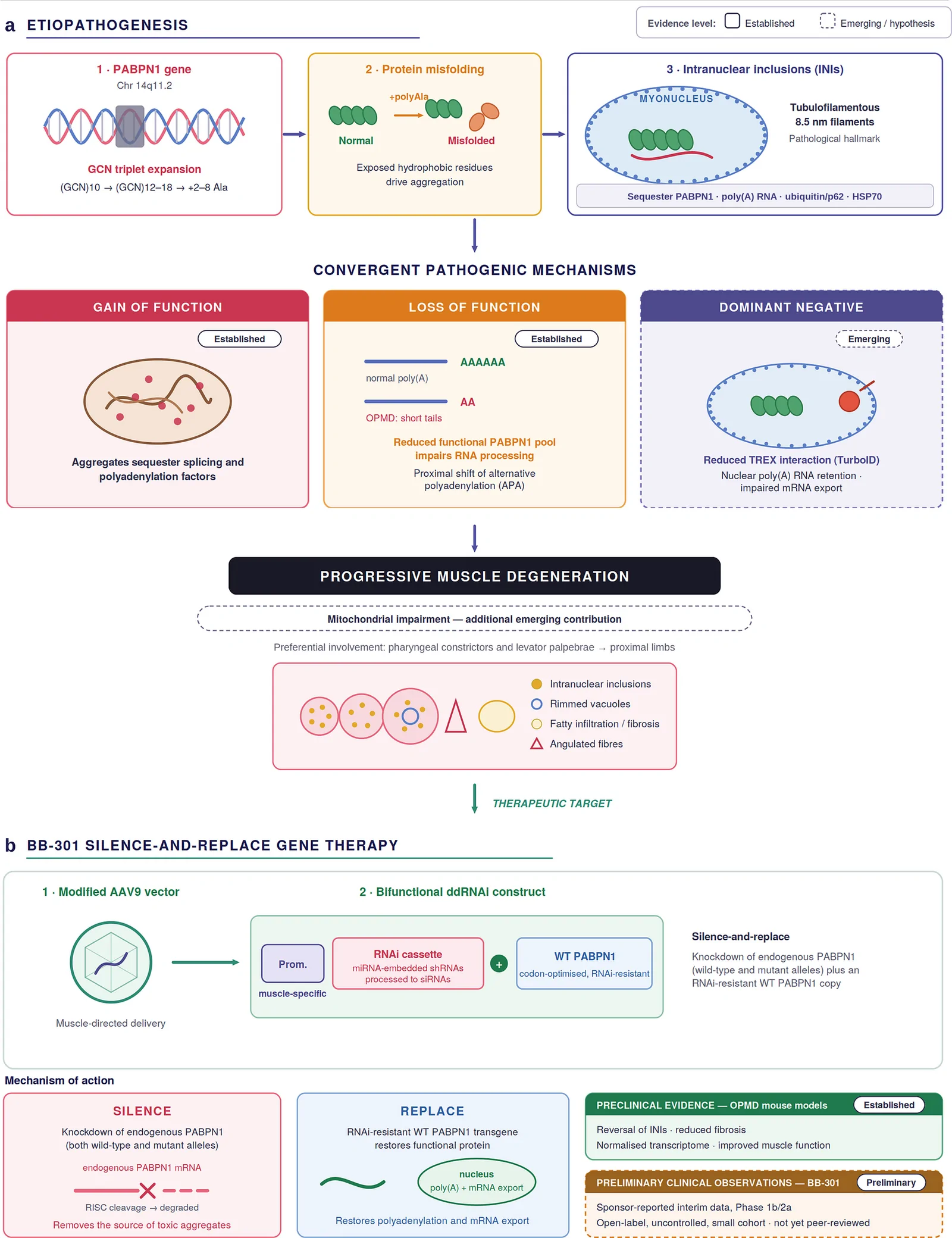
Pathogenic mechanisms of oculopharyngeal muscular dystrophy and rationale for the silence-and-replace gene therapy approach. **a** Etiopathogenesis. GCN-repeat expansion in PABPN1 (14q11.2) extends the N-terminal polyalanine tract from 10 to 12–18 residues. The range shown refers to fully penetrant alleles; the (GCN)11 allele is also pathogenic, with markedly reduced penetrance. The mutant protein misfolds and assembles into 8.5-nm tubulofilamentous intranuclear inclusions (INIs) that sequester PABPN1, poly(A) RNA, ubiquitin/p62, and HSP70. Mechanisms are shown according to the level of evidence. Established mechanisms include a gain of function, in which aggregates sequester splicing and polyadenylation factors, and a loss of function, characterised by a reduced functional PABPN1 pool, shortened poly(A) tails, and a proximal shift in alternative polyadenylation. An emerging mechanism is a dominant-negative effect involving reduced interaction with the TREX complex, nuclear retention of poly(A) RNA, and impaired mRNA export, as shown by TurboID. These convergent mechanisms drive progressive muscle degeneration, initially affecting the pharyngeal constrictors and levator palpebrae and subsequently the proximal limb muscles, with INI-bearing nuclei, rimmed vacuoles, fatty and fibrotic replacement, and angulated fibres. Mitochondrial impairment is represented as an additional emerging downstream contributor to progressive muscle degeneration rather than as a direct primary effect of polyalanine-expanded PABPN1. **b** BB-301 silence-and-replace gene therapy. A modified AAV9 delivers a single bifunctional ddRNAi construct in which a muscle-specific promoter drives the co-expression of an RNAi cassette, comprising miRNA-embedded shRNAs processed into siRNAs, and a codon-optimised, RNAi-resistant wild-type PABPN1 transgene. Both components are required: the RNAi cassette knocks down endogenous PABPN1, targeting both wild-type and mutant alleles rather than selectively silencing the mutant allele, whereas the resistant transgene restores functional PABPN1. In OPMD mouse models, this strategy clears INIs, reduces fibrosis, restores the transcriptome, and improves muscle function, providing preclinical proof of concept. Clinical evidence is currently limited to preliminary sponsor-reported interim observations from an ongoing open-label, uncontrolled Phase 1b/2a study that have not yet undergone peer review. Abbreviations: AAV9, adeno-associated virus serotype 9; APA, alternative polyadenylation; ddRNAi, DNA-directed RNA interference; INI, intranuclear inclusion; PABPN1, poly(A)-binding protein nuclear 1; RISC, RNA-induced silencing complex; TREX, transcription-export complex; WT, wild-type

The most advanced therapeutic approach is gene therapy based on the silence-and-replace principle. Malerba and colleagues showed that treatment of the Ala17 mouse model with two AAV vectors, one carrying a tricistronic shRNA cassette (delivered via AAV8) targeting endogenous *Pabpn1*, and one expressing a sequence-optimised replacement gene (delivered via AAV9) substantially reduced insoluble aggregates, decreased fibrosis, normalised muscle strength, and restored the muscle transcriptome [68]. Knockdown alone or replacement alone failed to provide comparable benefit, demonstrating the necessity of the dual approach. Follow-up studies confirmed that established INIs can be efficiently reversed by this strategy [69]. The subsequent consolidation of the silence-and-replace cassettes into a single dual-function AAV9 vector, which became BB-301, was described by Strings-Ufombah and colleagues [70] (Fig. 3).

### BB-301: First-in-Human Gene Therapy for OPMD

BB-301 (Benitec Biopharma) is the first and currently the only clinical-stage disease-modifying therapy for OPMD (NCT06185673) [15]. The drug is based on a DNA-directed RNA interference approach using a modified AAV9 capsid. BB-301 is a single construct that combines endogenous *PABPN1* knockdown with expression of a codon-optimised, RNAi-resistant wild-type PABPN1 replacement transgene [70]. The drug has been evaluated in a Phase 1b/2a trial (NCT06185673) [15] at a single site (NYU Langone Health, New York), with additional sites planned in Canada and France. BB-301 is directly injected into the pharyngeal constrictor muscles using an open surgical approach. The drug has been granted Orphan Drug designation by the FDA and EMA, as well as Fast Track status by the FDA (November 2025). As of mid-2026, Cohort 1, consisting of six patients at low dose, has been fully recruited and all six patients have completed 12-month follow-up. According to sponsor-reported interim analyses, improvements in swallowing-related outcome measures were reported to be sustained at 12 months and, in the first treated patient, at 24 months. Cohort 2 at high dose has begun recruitment, with the first two patients treated. Clinical data on Cohort 1 and Cohort 2 referenced throughout this section derive from sponsor press releases and the ClinicalTrials.gov registry entry (NCT06185673) [15]; no peer-reviewed trial results are available at the time of writing, and these observations are therefore preliminary.

### Other Emerging Approaches

CRISPR/Cas9 gene editing offers the potential for permanent correction of the GCN repeat expansion, though challenges in delivery efficiency and precision in post-mitotic muscle fibres remain [71, 72]. Antisense oligonucleotides (ASOs) and transcriptional repression using catalytically inactive Cas9 (dCas9) represent alternative strategies for suppressing mutant PABPN1 expression. The advantage of ASOs lies in their potential for repeated dosing and dose titration, while the disadvantage is the need for chronic administration, in contrast to the one-time dosing paradigm of AAV-based gene therapy [73, 74]. However, the therapeutic application of conventional ASOs to neuromuscular disorders is limited by inefficient delivery to skeletal muscle. Peptide–oligonucleotide conjugates, particularly those incorporating cell-penetrating peptides, can substantially enhance oligonucleotide uptake and activity in skeletal muscle compared with unconjugated oligonucleotides, as demonstrated in preclinical models of myotonic dystrophy [75]. Such delivery strategies may therefore increase the therapeutic potential of ASO-based approaches for OPMD, although their efficacy in OPMD-specific models remains to be established.

Muscle-enhancing approaches may complement gene therapy to address the downstream effects of muscle wasting. Inhibiting myostatin with antibodies, soluble receptors, or overexpression of follistatin has the advantage of enhancing muscle mass and counteracting muscle atrophy [76]. Modulating the IGF-1/mTOR pathway and the ubiquitin–proteasome pathway may address the metabolic and proteostatic defects. Inhibiting the FOXO pathway and anti-fibrotic therapy to address fibro-fatty replacement of muscle may be emerging therapeutic options.

Agents that enhance aggregate clearance, like trehalose, a natural disaccharide that stimulates autophagy, have been effective in clearing aggregates and slowing disease progression in the Ala17 mouse model [77]. Trehalose, administered intravenously, has progressed to clinical trials, which have shown promising open-label results in the treatment of OPMD, but development was not pursued further after the sponsoring company encountered financial difficulties. The phase 2/3 clinical study of intravenous trehalose in ALS, as part of the HEALEY ALS Platform Trial, did not achieve its primary outcomes [78], demonstrating the difficulties of pharmacological treatment that does not directly address the underlying pathology of the disease. Autologous myoblast transplantation has been tested in a phase I/IIa clinical study with modest results, as the concurrent cricopharyngeal myotomy may have masked any effect attributable to the grafted cells [79]. Xenograft models confirmed that PABPN1 aggregates reappear after muscle regeneration, limiting the durability of cell-based approaches [35]. Transcriptional blockade of the expanded repeat by catalytically inactive Cas9, which impedes transcription without cleaving DNA, has been demonstrated in other repeat expansion disorders but has not yet been applied to OPMD [80]. Doxycycline, whose anti-aggregation properties attenuated and delayed the toxicity of the polyalanine expansion in transgenic OPMD mice, was explored preclinically but did not progress to the clinic [81]. Combinatorial strategies integrating gene therapy with muscle-enhancing or anti-aggregate approaches may ultimately provide the most comprehensive therapeutic benefit (Table 1).

**Table 1:** Current and emerging therapeutic strategies for oculopharyngeal muscular dystrophy (OPMD). Clinical findings reported for BB-301 are derived from sponsor-reported interim analyses and ClinicalTrials.gov records. Peer-reviewed clinical trial data are not yet available

Therapeutic approach	Mechanism of action	Development stage	Key results/evidence	Limitations/challenges	References
***Gene therapy***					
BB-301 (Benitec Biopharma)^a^	AAV9-based ddRNAi silence-and-replace: RNAi cassette (miRNA-embedded shRNAs) silences endogenous *PABPN1* + codon-optimised, RNAi-resistant WT *PABPN1* replacement	Phase 1b/2a (NCT06185673); FDA Orphan Drug + Fast Track; EMA Orphan Drug	Sponsor-reported interim findings indicated that all treated participants met the study-defined responder criteria; Cohort 1 (low dose, n = 6): sustained improvement at 24 months (Patient 1); restored swallowing reportedly within normal limits (Patients 2–3); pathologic sequential swallows reduced from 80% to 17% (Patient 2, thin liquid). Cohort 2 (higher dose): initiated Q4 2025, first two patients treated by May 2026, early data suggest improved depth of response	Intramuscular injection into pharyngeal constrictors (open surgical approach); does not address limb weakness; long-term durability data awaited; controlled data pending. Data derived from company press releases; peer-reviewed and controlled clinical data not yet available	Clinical (ongoing): [15]. Vector design: [70]
Silence-and-replace (preclinical concept)	AAV-mediated tricistronic shRNA knockdown of endogenous *Pabpn1* + sequence-optimised replacement gene	Preclinical (Ala17 mouse model)	Malerba et al. [68] (two-vector approach); reversibility of established INIs demonstrated in Malerba et al. [69] (two-vector); subsequently consolidated into a single dual-function AAV9 vector as BB-301 (Strings-Ufombah et al. [70])	Transgenic overexpression model may not fully recapitulate human disease; translation to clinical dosing required optimisation	Preclinical: [68–70]
***Gene editing and RNA-targeted approaches***					
CRISPR/Cas9	Direct correction of GCN expansion in *PABPN1* exon 1; potential for permanent genomic repair	Conceptual	Proof-of-concept in other repeat expansion disorders with no OPMD-specific therapeutic studies available	Delivery efficiency to post-mitotic skeletal muscle; off-target editing; GC-rich repeat region poses technical challenges; immunogenicity of Cas9	[71, 72]^c^
Antisense oligonucleotides (ASOs) and peptide–ASO conjugates	Targeted degradation of mutant *PABPN1* mRNA; allele-selective silencing potentially achievable; conjugation to cell-penetrating peptides to enhance skeletal muscle uptake and intracellular delivery	Conceptual	Established platform in other neuromuscular disorders (e.g., nusinersen for spinal muscular atrophy, tofersen for SOD1-ALS). Peptide–oligonucleotide conjugates substantially enhance oligonucleotide uptake and activity in skeletal muscle compared with unconjugated oligonucleotides in preclinical models of myotonic dystrophy [75]. No OPMD-specific studies available	Requires chronic repeated administration; does not replace WT PABPN1 function; potential for off-target knockdown of WT allele; inefficient delivery of conventional ASOs to skeletal muscle, only partly mitigated by peptide conjugation, which remains unvalidated in OPMD models; delivery to pharyngeal muscle uncertain	[73–75]^c^
Transcriptional repression (dCas9)	Transcriptional blockade at the expanded repeat by catalytically inactive Cas9, without DNA cleavage	Conceptual	Demonstrated in other repeat expansion contexts. Application to OPMD remains theoretical	Delivery challenges; sustained expression of the dCas9 construct required; allele selectivity; immune response to bacterial Cas9 protein	[80]^c^
***Pharmacological approaches***					
Trehalose	Natural disaccharide; promotes autophagy-mediated clearance of protein aggregates	Phase 2 and Phase 3 (OPMD); Phase 2/3 (ALS); discontinued	Reduced aggregate burden and delayed progression in Ala17 mouse model [77]. Encouraging open-label Phase 2 results in OPMD. Phase 2/3 HEALEY Platform Trial in ALS failed primary endpoints	Development discontinued due to sponsor financial difficulties (OPMD) and lack of efficacy in ALS; does not address underlying genetic defect; IV administration	Preclinical: [77]. Clinical: [78]^b^
Doxycycline	Anti-aggregation properties; attenuates PABPN1 toxicity in transgenic models	Preclinical only	Attenuated and delayed toxicity in transgenic OPMD mice [81]	Non-specific mechanism; long-term antibiotic use concerns; modest effect size; no clinical translation	Preclinical: [81]
***Muscle-boosting and supportive strategies***					
Myostatin inhibition	Blockade of myostatin signalling (antibodies, soluble receptors, or follistatin overexpression) to enhance muscle mass	Preclinical	Proven muscle hypertrophy in preclinical models of other neuromuscular disorders. Improvement of muscle atrophy demonstrated in the Ala17 OPMD mouse model [76]. Clinical efficacy data in OPMD are currently lacking	Does not address *PABPN1* mutation or aggregation; mixed clinical results in other dystrophies; potential off-target effects	Preclinical (OPMD-specific): [76]
IGF-1/mTOR pathway modulation	Enhancement of anabolic signalling to counteract muscle atrophy and metabolic deficits	Conceptual	Rationale based on transcriptomic evidence of insulin signalling dysregulation in OPMD muscle [59]	Risk of promoting fibrosis; metabolic side effects; does not target primary defect	[59]^c^
Anti-fibrotic strategies	Prevention/reversal of fibroadipose replacement of muscle tissue	Conceptual	Fibrosis is a prominent feature in OPMD muscle and Ala17 model; reversal demonstrated with silence-and-replace gene therapy	Secondary to disease process; unlikely to provide benefit as monotherapy; biomarker development needed	[39, 68, 76]^c^
***Cell-based therapy***					
Autologous myoblast transplantation	Injection of patient-derived myoblasts into pharyngeal muscles to restore functional muscle tissue	Phase I/IIa (completed)	Modest clinical results, as the concurrent cricopharyngeal myotomy may have masked any effect attributable to the grafted cells (Perié et al. [79])	PABPN1 aggregates reappear after regeneration (Roth et al. [35]); technical complexity of pharyngeal delivery; limited durability	Clinical (Phase I/IIa): [79]. Preclinical durability data: [35]

*AAV* adeno-associated virus, *AAV9* adeno-associated virus serotype 9, *ALS* amyotrophic lateral sclerosis, *ASO* antisense oligonucleotide, *dCas9* catalytically inactive Cas9, *ddRNAi* DNA-directed RNA interference, *EMA* European Medicines Agency, *FDA* US Food and Drug Administration, *IGF-1* insulin-like growth factor 1, *INI* intranuclear inclusion, *miRNA* microRNA, *mTOR* mechanistic target of rapamycin, *PABPN1* poly(A)-binding protein nuclear 1, *RNAi* RNA interference, *shRNA* short hairpin RNA, *siRNA* small interfering RNA, *WT* wild-type

^a^Clinical findings reported for BB-301 derive from sponsor-reported interim analyses and from the ClinicalTrials.gov registry entry (NCT06185673): Benitec Biopharma press releases of 19 March 2025, 3 November 2025, 11 January 2026, 9 March 2026 and 14 May 2026; interim results presented at the MDA Clinical & Scientific Conference (poster 501 LB, 9 March 2026) and at the ASGCT Annual Meeting (oral presentation, 15 May 2026). None of these sources has undergone peer review, and controlled clinical data are not yet available

^b^The open-label Phase 2 (NCT02015481) and pivotal Phase 3 (NCT02328482) trials of intravenous trehalose in OPMD have not been reported in a peer-reviewed publication. Reference [78] refers to the Phase 2/3 HEALEY ALS Platform Trial

^c^No OPMD-specific therapeutic studies are available. The cited references provide proof of concept in other repeat-expansion or neuromuscular disorders, or the biological rationale on which the proposed strategy is based

## Conclusions and Future Perspectives

Oculopharyngeal muscular dystrophy is entering a new era of scientific understanding and therapeutic opportunity. The recognition that pathogenesis involves convergent gain-of-function, loss-of-function, and dominant-negative mechanisms has provided the rational foundation for the silence-and-replace gene therapy approach. Sponsor-reported interim findings from the ongoing Phase 1b/2a study suggest potential clinical benefit and support further investigation of BB-301. However, these observations derive from a small open-label uncontrolled study and require confirmation through peer-reviewed analyses and controlled clinical trials. Several issues remain to be addressed. Non-invasive biomarkers such as circulating miRNAs, quantitative MRI, and outcome measurements of swallowing will be critical for efficient study design. Systemic gene therapy to address the weakness of the limbs will be a future objective. Increasing clinician awareness of the existence of OPMD, which will be helped by genetic testing and muscle biopsy PABPN1 immunofluorescence, will be critical to reduce diagnostic delay. The epidemiological data available suggest that the true prevalence of OPMD may be greater than that traditionally cited, especially in populations without a recognised founder effect. The implications of success extend beyond OPMD itself. The silence-and-replace approach represents a proof of concept applicable to other autosomal dominant diseases involving toxic gain-of-function and loss-of-function, a paradigm relevant to conditions as diverse as certain forms of amyotrophic lateral sclerosis, myotonic dystrophy, and dominant retinitis pigmentosa. The technical innovations in delivering gene therapy to pharyngeal muscles, the development of swallowing-specific outcome measures, and the integration of natural history data into clinical trial design contribute methodological tools to the broader neuromuscular disease community.

Looking further ahead, the convergence of gene therapy with complementary approaches, including pharmacological enhancement of proteostasis, myostatin inhibition, and anti-fibrotic strategies, offers the prospect of combinatorial treatments tailored to disease stage and individual patient needs. The integration of artificial intelligence into multi-omics analysis and clinical trajectory modelling may enable personalised therapeutic decisions. For patients with OPMD, including those from populations without a recognised founder effect, the emergence of disease-modifying therapeutic strategies represents an important turning point. The journey from Taylor’s first description in 1915 to BB-301’s entry into clinical testing in 2024 spans over a century, but the pace of discovery has accelerated dramatically, raising the possibility that emerging disease-modifying therapies may improve the management of OPMD in the future, pending confirmation in larger controlled studies.

## Data Availability

No datasets were generated or analysed during the current study.

## Abbreviations

AAV: Adeno-associated virus
AAV8: Adeno-associated virus serotype 8
AAV9: Adeno-associated virus serotype 9
ALS: Amyotrophic lateral sclerosis
APA: Alternative polyadenylation
ASO: Antisense oligonucleotide
COX: Cytochrome c oxidase
dCas9: Catalytically inactive Cas9
ddRNAi: DNA-directed RNA interference
DIGEST: Dynamic Imaging Grade of Swallowing Toxicity
EMA: European Medicines Agency
ER: Endoplasmic reticulum
FDA: US Food and Drug Administration
FISH: Fluorescence in situ hybridization
FOXO: Forkhead box O
FRAP: Fluorescence recovery after photobleaching
FTD: Frontotemporal dementia
IGF-1: Insulin-like growth factor 1
INI: Intranuclear inclusion
miRNA: MicroRNA
MRI: Magnetic resonance imaging
mTOR: Mechanistic target of rapamycin
NGS: Next-generation sequencing
NRRSv: Normalised residue ratio scale, valleculae
OPDM: Oculopharyngodistal myopathy
OPMD: Oculopharyngeal muscular dystrophy
PABPN1: Poly(A)-binding protein nuclear 1
RNAi: RNA interference
SDH: Succinate dehydrogenase
shRNA: Short hairpin RNA
siRNA: Small interfering RNA
SSQ: Sydney Swallow Questionnaire
TDP-43: TAR DNA-binding protein 43
TPR: Total pharyngeal residue
TREX: Transcription-export complex
VFSS: Videofluoroscopic swallowing study
WT: Wild-type
